# A longitudinal replication study testing migration from video game loot boxes to gambling in British Columbia, Canada

**DOI:** 10.1186/s40359-025-02766-1

**Published:** 2025-04-30

**Authors:** Lucas Palmer, Gabriel A. Brooks, Luke Clark

**Affiliations:** 1 Department of Psychology, Centre for Gambling Research at UBC, University of British Columbia, Vancouver, B.C Canada; 2 Djavad Mowafaghian Centre for Brain Health, University of British Columbia, Vancouver, B.C Canada

**Keywords:** Loot boxes, Gambling behavior, Randomized rewards, Behavioral addiction, Replication, Bayesian, Longitudinal

## Abstract

**Background:**

Loot boxes are randomized reward mechanics in modern video games that share features with conventional gambling products. Research studies have begun to test longitudinal patterns (“migration”) from engagement with loot boxes to gambling behavior. This study investigated such effects at a 6-month follow-up in an online sample of young adults that play video games (aged 19—25) from British Columbia, Canada.

**Methods:**

Participants were stratified into two subgroups at their baseline assessment: 83 reported they did not currently gamble and 43 reported they currently gamble, after cleaning. At baseline, participants provided responses to the Risky Loot Box Index (RLI) and estimates of their past year spending on both randomized (i.e., loot boxes) and non-randomized (“direct purchase”) microtransactions. Microtransaction spending and RLI scores at baseline were tested as predictors of self-identified gambling initiation and spend at follow-up. We tested a set of frequentist regressions and a corresponding set of Bayesian regressions.

**Results:**

At baseline, participants who reported gambling showed higher levels of engagement with both randomized and non-randomized microtransactions. Among non-gambling participants at baseline, loot box spending and RLI predicted gambling initiation at the follow-up, in a Bayesian logistic regression with informed priors. Loot box spending and RLI at baseline predicted gambling expenditure at follow-up, in both the frequentist and Bayesian linear regressions. Spending on direct purchase microtransactions did not predict gambling initiation in either set of models when controlling for loot box spending, underscoring the role of randomized rewards.

**Conclusions:**

These data provide further prospective evidence for gambling ‘migration’ in a sample recruited in Western Canada, indicating that young adults who spend money on loot boxes are at elevated risk for real-money gambling.

## Background

‘Loot boxes’ are a common feature of modern video games in which randomized prizes can be purchased or earned through video game play [1, 2]. Many video games also include non-randomized microtransactions, such as buying a specific virtual item from an in-game store, which we term “direct purchase” microtransactions (DPMs) [3–5]. A growing body of evidence supports the linkages between loot box purchasing, gambling engagement, and problematic gambling [6–8]. Consumption of loot boxes is also associated with wider impacts on wellbeing, such as lower educational attainment and greater psychological distress [9]. As such, loot boxes are the subject of increasing concern from a public health perspective, raising questions around how these gambling-like products should be regulated [10].

Evidence supporting the link between loot box use and gambling was initially based on cross-sectional studies that indicated positive correlations between measures of problem gambling and loot box involvement. These studies either recorded spending on loot boxes, or scores on self-report measures such as the Risky Loot Box Index (RLI), a 5 item scale assessing risky and ‘compulsive’ use of loot boxes [5, 6, 11]. For example, in a meta-analysis, the association between loot box spending and problem gambling was robust, with a small-to-moderate effect size (*r* = 0.260) [12]. Other studies have observed moderate associations between RLI scores and problem gambling severity [6, 13]. Many researchers have taken care to note that cross-sectional data do not speak directly to any causal relationship between loot boxes and gambling [14, 15]. It is possible that loot boxes may promote future gambling, such as by exposure and ‘sensitization’ to unpredictable reward schedules [16], but it is also possible that when people who are experienced with gambling play video games, they are drawn to loot box features, based on their familiarity with randomized monetary rewards [5].

Longitudinal research can help to disentangle these potential pathways. A recent study by our team tested the ‘migration’ from loot box use to gambling in young adults aged 18 to 25, recruited online using the Prolific platform [3]. This was a geographically diverse sample from the UK, North America and Australia. Increased spending on loot boxes—but not DPMs—at baseline predicted an increased likelihood of initiating conventional gambling at the 6-month follow-up. Further, there was a positive association between loot box spending at baseline and spending on conventional gambling at the 6-month follow-up. This study also observed that RLI scores at the baselines survey positively predicted both migration and spending on conventional gambling at the follow-up. These temporal relationships were corroborated in a second study by González-Cabrera et al., [17] in a sample of Spanish adolescents, in whom baseline loot box engagement significantly increased engagement with online gambling and symptoms of problem gambling at a 6 month follow up.

The speed with which loot box mechanics were introduced into the modern video game landscape has left a notable gap in regulation and treatment provision, with consequences for consumers. As a regional example, in the province of British Columbia (BC), Canada, this knowledge gap has raised at least two pertinent questions: 1) whether video game loot boxes constitute a form of unregulated gambling, and 2) whether a dedicated counselling program for disordered gambling can or should provide care for people experiencing financial harms from gambling-like elements of video games. The regulatory question is reflected in an ongoing class action lawsuit against the video game company Electronic Arts, which cites the operation of unlicensed illegal gambling systems and deceptive practices via the offering of “vanishingly small odds” for potential prizes [18]. With respect to the question surrounding treatment provision, excessive engagement with video gaming, i.e., gaming disorder, was formally recognized by the World Health Organization in 2019, and many jurisdictions continue to lack clinical expertise in this area. The excessive consumption of loot boxes can lead to a pattern of harms that are primarily financial [19], and thus may overlap more with the clinical profile of gambling disorder (e.g., loss chasing, borrowing money) than the typical phenomenology of excessive video gaming (e.g., social withdrawal, impaired sleep). In BC, where a provincial counselling program for gambling problems (GamblingSupportBC: https://www.gamblingsupportbc.ca/) operates outside of the standard healthcare system, how should services accommodate youth or adults experiencing harm from loot box mechanics of video gaming?

The present study was designed as a replication of the longitudinal study by Brooks & Clark [3], in a sample restricted to BC, in order to build provincial (i.e., jurisdictional) knowledge around the convergence of gambling and gaming. Replication is an essential step in the scientific process [20]. Researchers can assess the degree of certainty surrounding a particular effect, as evidence accumulates across multiple studies using similar or identical designs [21]. The sample for this study focuses on young adults aged 19–25, as an age window characterized by high involvement in video gaming, and we reasoned that (legal) initiation of gambling as our target variable would increase in incidence during this period [22]. Alongside analyses corresponding to those reported in Brooks & Clark [3], we also incorporate a series of Bayesian analyses to test support for the null hypothesis, due to the relatively smaller sample size of the current study, and using priors informed by the Brooks & Clark data [3]. Specifically, we test four hypotheses: H1) at baseline, loot box purchasing, but not DPM purchasing, predicts later initiation of gambling at 6-month follow-up, H2) at baseline, RLI scores predict later initiation of gambling at 6-month follow-up, H3) at baseline, loot box purchasing, but not DPM purchasing, predicts gambling spend at 6-month follow-up, H4) at baseline, RLI scores but not DPM purchasing, predicts gambling spend at 6-month follow-up.

## Methods

### Sample & procedure

We used two approaches to recruit young adults from local online communities in British Columbia. First, using Reddit, we advertised on local subreddits. Second, we advertised on a Discord server for eSports at a University in Western Canada. We contacted moderators for these boards to obtain permission for posting our study advertisement. The Prolific platform that was used for recruitment in Brooks & Clark [3] was not a viable route for the present study because it does not enable targeted recruitment to a specific Canadian province. Respondents were directed to Qualtrics for a pre-screen survey (accessed by *n* = 1,465) to establish the following eligibility criteria:

- age range 19 (as the minimum legal for gambling in BC) to 25.
- regular video gaming, defined as ≥ 3 h per week
- resident in BC
- proficient in English
- Endorsing familiarity with loot box mechanics based on the following definition: “Loot boxes” have become a prominent feature in video games over the past decade. Loot boxes can be earned through gameplay (as an occasional reward or by spending accumulated in-game points) or, in recent games, may be purchased directly. When a loot box is opened, the player receives a randomly generated virtual item.”

Based on the pre-screen responses, we stratified participants into two groups based on their response to the item “Do you currently gamble?”, with participants who answered ‘no’ classified as *Non-gambling* and those who answered ‘yes’ as *Gambling*. The pre-screen took less than 5 min to complete and was not reimbursed. Eligible participants (*n* = 977) then received an email invitation and link to the baseline assessment on Qualtrics, titled ‘Video Game Spending – Part 1’, which took approximately 35 min to complete. *N* = 317 participants completed the baseline survey and were contacted by email after 6 months to participate in a follow-up survey titled ‘Video Game Spending – Part 2’ which took approximately 10 min to complete. Of those participants contacted for follow-up *n* = 172 completed the survey, indicating an attrition rate of 45.7%.

Three stages of cleaning were applied to this dataset to ensure data quality following the same set of exclusion criteria applied in Brooks & Clark [3], with the addition of participant indication of incorrect recruitment platform, which was included in this study over concerns of data quality in sampling from Reddit. In stage 1, the eligibility criteria on the pre-screen were re-administered in the baseline assessment, and participants who made discrepant responses were removed; namely 1) three participants were not current video gamers, 2) two participants were not comfortable with English, 3) 20 participants indicated their residence as outside of BC, 4) six participants were outside of the required age range. In stage 2, a number of attention checks were applied to the baseline survey dataset (recommended by Goodman et al., [23]): 1) consistent entry of the participant’s age across the pre-screen and baseline assessment; 2) did not endorse playing a fictional slot machine; 3) did not endorse playing a fictional video game; and 4) selection of a specified answer for a question. Of these items, 87 participants were excluded for failing more than 1 attention check. In stage 3, we checked for quality and consistency in participants’ responses across their baseline dataset: 1) 8 participants had inconsistent responses across the survey (e.g., answered ‘Yes’ to currently gambling but ‘No’ to ever having gambled), 2) 60 were removed for indicating an incorrect recruitment platform (i.e., stating they were recruited from either mTurk or Prolific), as such responses were likely to have come from bots, 3) 5 participants were removed for excessive length in completing survey (3SD over the median survey length). This left us with 126 participants for the baseline survey. The sample size reduction of 60% was notably larger than the equivalent rate in Brooks & Clark [3] of 11%. This stark difference likely reflects the disparity in data quality between recruiting from research focused crowdsourcing platforms such as Prolific relative to social media websites such as Reddit.

The pre-screen, baseline assessment, and follow-up survey all involved informed consent to proceed. Study approval was provided by the University of British Columbia Ethics Board. The pre-screen and baseline assessment were launched and completed in July 2021, with the 6-month follow-up run in January 2022. Participants were reimbursed $8.50 for completing the baseline assessment and $4.50 for the follow-up survey. Reimbursements were made using the Tango Cash rewards program, which provides an online gift card that can be redeemed at a variety of online stores (e.g., Amazon, Apple, Starbucks), delivered to the participant’s email address.

### Measures

The baseline survey included general demographics measures (age, gender, ethnic background), measures of video gaming engagement including microtransactions, and gambling engagement. Specifically, participants reported their video game engagement (hours per week); estimated age of starting video gaming; exposure to, and engagement with, loot boxes and DPMs; and expenditure on loot boxes and DPMs over the previous 12 months. Participants reported whether they currently gambled, and whether they had gambled at all in the past.

On the baseline survey, participants completed the Risky Loot Box Index (RLI), a five-item single factor scale that assesses perceived risky and compulsive use of loot boxes developed by Brooks & Clark [6]. The index includes questions such as “I frequently play games longer than I intend to, so I can earn Loot Boxes”, and “Once I open a Loot Box, I often feel compelled to open another”. In our baseline sample the RLI demonstrated acceptable internal consistency (α = 0.716).

The two main variables for our longitudinal analysis were whether the participant self-reported as a currently gambling at the follow-up (T2), and the estimated amount of money participants spent on conventional (i.e., non-loot box) gambling at follow-up over the previous 6-months.

### Analysis plan

In Brooks & Clark [3], a pre-registration plan was submitted for the migration hypotheses, data cleaning strategy, and the target sample size of 392 per group. In the present study, we were unsure about the feasibility of recruiting a comparable number of participants, within BC and using the more purposive sampling. Hence, the present study is described as exploratory, although our frequentist analyses reproduce tests that were pre-registered in Brooks & Clark [3]. We conduct a logistic regression to assess whether loot box engagement (continuous) and the risky use of loot boxes (RLI score, continuous) predict gambling initiation (dependent variable 1) at the 6-month follow-up assessment. We use linear regression to assess whether loot box engagement and RLI score at baseline predicts gambling spend (dependent variable 2) at follow-up. These inferential analyses are performed only on the non-gambling group at baseline, though we report comparisons between the gambling and non-gambling groups at baseline for group characterization. As the regression analyses entail four models, we adjusted our alpha from *p* < 0.05 to *p* < 0.0125. As the expenditure variables were skewed (as in Brooks & Clark [3]), we employed the same log base 2 + 1 transform to improve normality, with base 2 being used to facilitate interpretability of odds ratios (i.e., reflecting a change in odds per doubling of expenditure). Analyses were conducted in R version 4.3.2 [24], using the following packages: aod [25], DescTools [26], and stats [24].

Given the smaller sample in the current study, we also conducted a series of Bayesian analyses to address some limitations inherent to frequentist statistics. Bayesian statistics can quantify the degree of support for the alternative hypothesis relative to the null hypothesis through the computation of Bayes Factors (BF_10_) [27]. Specifically, for each parameter in a Bayesian regression, the resulting BF_10_ indicates the degree of support for the alternative hypothesis over the null hypothesis. This is particularly useful when the null cannot be rejected using significance testing, which can depend on large sample approximations [28]. The convention for interpreting bayes factors is that a BF_10_ of 1 means there is no evidence for either the null or alternative hypothesis. Values greater than 1 present evidence in favour of the alternative hypothesis, and values less than 1 represent evidence in favour of the null hypothesis. Stefan et al. [29] describe a classification scheme for interpreting BF_10_: a BF_10_ of 1–3 represents anecdotal evidence for the alternative hypothesis, 3–10 is moderate evidence, 10–30 is strong evidence, 30–100 is very strong, and any value over 100 is extreme evidence in favour of the alternative hypothesis over the null. A further benefit of Bayesian methods is they allow researchers to incorporate background knowledge for a given effect using a probability distribution called the prior [30]. When information about model parameters is known from previous research (e.g., values of coefficients and standard errors), this information can be used to set informed priors. In the current study, we set informed priors for our analyses using data from Brooks & Clark [3].^1^

We conducted a series of four Bayesian regressions corresponding to the four frequentist tests outlined above. Analysis steps were the same for all models: first, we ran the regressions using uninformed broad priors to elicit Bayes Factors for comparison to the later models based on informed priors. This comparison is useful to demonstrate the sensitivity of the posterior distribution to the prior distribution, since small datasets can be more sensitive to the prior [28]. Next, we conducted Bayesian regressions with broad priors on data from Brooks & Clark [3], to obtain a posterior distribution that could be applied to create informed priors for each parameter in the model of the current data. Finally, we conduct the same Bayesian regressions on the current dataset as done in the first step, this time using the informed priors elicited from the posterior distribution of the Bayesian regressions of the Brooks & Clark dataset [3, 27]. We only include the results of the informed priors analysis in the results section, but the analysis code for the preceding steps is available in the supplemental material. The following R packages were used in the Bayesian analyses: bayestestR [31] brms [32], rstanarm [33].

## Results

### Baseline demographics and microtransaction descriptives

Table 1 shows the participant demographics in the two subgroups of non-gambling and gambling participants. The gambling participants at baseline were significantly older than the non-gambling group (*t*_98.73_ = 2.12, *p* = 0.037), despite the narrow age range for recruitment. The groups did not differ by gender (χ2 = 0.28, *p* = 0.871). Groups differed somewhat by ethnicity, with a greater proportion of European participants in the gambling group (86%) compared to a more mixed proportion of ethnicities in the non-gambling group. We also tested whether differences existed in baseline demographic variables between participants who did versus did not complete the follow-up. There was no significant difference in age (*t*_123.37_ = 0.95, *p* = 0.342) or gender (χ2 = 0.11, *p* = 0.944), but a difference was observed on ethnicity (χ2 = 20.64, *p* = 0.944), which was likely driven by a greater proportion of East Asian participants in the follow-up group.

**Table 1:** Participant demographics of the two subgroups at the baseline assessment

	Non-gambling (*n* = 83)	Gambling (*n* = 43)
N at follow-up (%)	*n* = 54 (65.1%)	*n* = 20 (46.5%)
Demographic Variable		
Mean Age (SD)	21.7 (1.75)	22.3 (1.48)
Gender:		
men (%)	63 (75.9)	33 (76.7)
women	19 (22.9)	9 (20.9)
non-binary	1 (1.2)	1 (2.3)
Ethnicity:		
African/Black (%)	0	1 (2.3)
Asian	4 (4.8)	0
East Asian	32 (38.6)	3 (7)
European	37 (44.6)	27 (86)
Middle Eastern	1 (1.2)	0
South East Asian	5 (6)	0
South Asian	3 (3.6)	0
Mixed East Asian/Southeast Asian	1 (1.2)	0
Mixed European/South East Asian	1 (1.2)	0
Mixed European/Asian	0	1 (2.3)
Mixed European/Indigenous	0	1 (2.3)
Other (Mixed)	1 (1.3)	0
Ever Gambled (%)	35 (45.8)	43 (100)

The baseline sample was sorted by participants’ current gambling status. Gambling status was determined by response (Yes/No) to the question, “Do you currently gamble?”

Engagement with loot box and DPM features as a function of baseline gambling status is presented in Table 2. Gambling participants reported higher spending on loot boxes (*z* = − 4.90, *p* < 0.001) and on DPMs (*z* = − 3.97, *p* < 0.001) compared to the non-gambling participants. Gambling participants were more likely to have bought loot boxes (χ2 = 8.24, *p* = 0.004), and sold loot box items (χ2 = 4.66, *p* = 0.031), relative to the non-gambling participants. We also compared the two subgroups on microtransaction involvement. Of the variables reported in Table 2, three of these comparisons were significant: participants who did not complete the follow-up were more likely to report selling an item (82.7% versus 59.5%; X2 = 7.71, *p* = 0.005), reported a higher spend on loot boxes (Median = $100 versus $50, *z* = − 2.28, *p* = 0.022) and reported a higher spend on DPMs (Median = $180, versus $ 80, *z* = − 2.47, *p* = 0.013).

**Table 2:** Loot box and DPM descriptives by gambling status at baseline

Variables	Non-gambling (n = 83)	Gambling (n = 43)	Test statistic
Video gaming per week	"About 26–30 h per week"	"About 26–30 h per week"	χ2 = 5.15, p =.881, φ =.202
Age started gaming (SD)	9.67 (3.84)	12.16 (3.48)	χ2 = 36.97, p =.002, φ =.542
Loot Boxes			
Familiar with… (%)	78 (94.0%)	39 (90.7%)	χ2 = 0.46, *p* =.498, φ =.060
Played a game with…	78 (94.0%)	39 (90.7%)	χ2 = 0.04., *p* =.835, φ =.019
Bought …	56 (67.5%)	39 (90.7%)	χ2 = 8.24, *p* =.004, φ =.256
Sold an item won in a loot box	52 (62.7%)	35 (85.4%)	χ2 = 4.66, *p* =.031, φ =.192
Past year spending on…	$15	$240	*U* = 840.5, z = − 4.90, *p* <.001, *r* =.437
DPMs			
Familiar with…	73 (88.0%)	40 (93.0%)	χ2 = 0.79, *p* =.375, φ =.079
Played a game with…	73 (88.0%)	39 (90.1%)	χ2 = 0.22, *p* =.641, φ =.041
Bought…	67 (80.7%)	39 (90.1%)	χ2 = 3.35, *p* =.067, φ =.163
Sold an item purchased as a DPM	37 (44.5%)	36 (83.7%)	χ2 = 17.81, *p* <.001, φ =.376
Past year spending on…	$70	$280	*U* = 1014, z = − 3.97, *p* <.001, *r* =.354

Spending data are medians, and were analyzed with Mann–Whitney U tests with a derived *r* value for effect size (interpretive range: 0.1 to 0.3 is small effect, 0.3 to 0.5 a moderate effect, 0.5 to 1.0 a strong effect). Group differences on categorical variables were assessed with chi-squared tests with phi (φ) for effect size (interpretive range: 0.0 to 0.1 is a negligible effect, 0.1 to 0.2 is weak, 0.2 to 0.4 is moderate, 0.4 to 0.6 is relatively strong, 0.6 to 0.8 is strong, and 0.8 to 1.0 is very strong; Rea et al., 2016)

### Frequentist analysis

#### Follow-up migration to gambling

For the longitudinal tests of migration, 54 (65.1%) of the non-gambling participants returned at 6 months (T2). Of these participants, 7 reported gambling initiation at the follow-up. The logistic regressions assessed whether the baseline predictors of DPM spending, loot box spending, or RLI scores predicted migration at follow-up. A hierarchical regression analysis was conducted, in which DPM spending was entered as a single predictor at Step 1, and loot box spending was added along with DPM spending at Step 2 to examine its incremental contribution (see Table 3). At step 1, DPM spending did not significantly predict migration (OR = 1.14, *p* = 0.277), or when added simultaneously with loot box spending at step 2 (OR = 0.90, *p* = 0.567). The predictive link between loot box spending at baseline and gambling migration, when controlling for baseline DPM spending, also failed to reach significance (OR = 1.31, *p* = 0.118). Lastly, the RLI scores at baseline did not significantly predict follow-up migration to current gambling status (OR = 2.11, *p* = 0.124).

**Table 3:** Logistic regressions predicting migration to gambling status (at T2) among baseline non-gambling participants

Microtransaction Expenditure							
**Variables Step 1**	**B**	**98.75% CI**	**SE**	**Wald**	**p-val**	**OR**	**98.75% OR CI**
Constant	− 2.63	− 5.27, − 0.81	0.86	9.3	.002		
DPM spending	0.13	− 0.17, 0.47	0.12	1.2	.277	1.14	0.84, 1.60
Test of Model Coefficient	χ2 = 4.8						
Cox & Snell/Nagelkerke R2	.023/.044						
**Variables—Step 2**	**B**	**98.75% CI**	**SE**	**Wald**	**p-val**	**OR**	**95% OR CI**
Constant	− 2.62	− 5.15, − 0.85	0.83	10.0	.002		
DPM spending	-.11	− 0.63, 0.38	0.19	.33	.567	.90	0.53, 1.46
Loot Box spending	.27	− 0.11, 0.78	0.17	2.4	.118	1.31	0.90, 2.20
Test of Model Coefficient	χ2 = 6.36						
Cox & Snell/Nagelkerke R2	.074/.138						
***Risky Loot Box Index***							
**Variables**	**B**	**98.75% CI**	**SE**	**Wald**	**p-val**	**OR**	**95% OR CI**
Constant	− 1.99	− 3.38, -.1.02	0.45	19.6	.001		
RLI Score	.75	− 0.34 2.18	0.48	2.4	.124	2.11	0.89, 6.22
Test of Model Coefficient	χ2 = 6.36						
Cox & Snell/Nagelkerke R2	.051/.094						

Significance level of *p* ≤.0125 and 98.75% CI are required for the Bonferroni correction applied. Expense variables were log base 2 + 1 transformed to reduce positive skew. RLI scores were standardized

#### Follow-up linear regressions on gambling expenditure

**Fig. 1:**
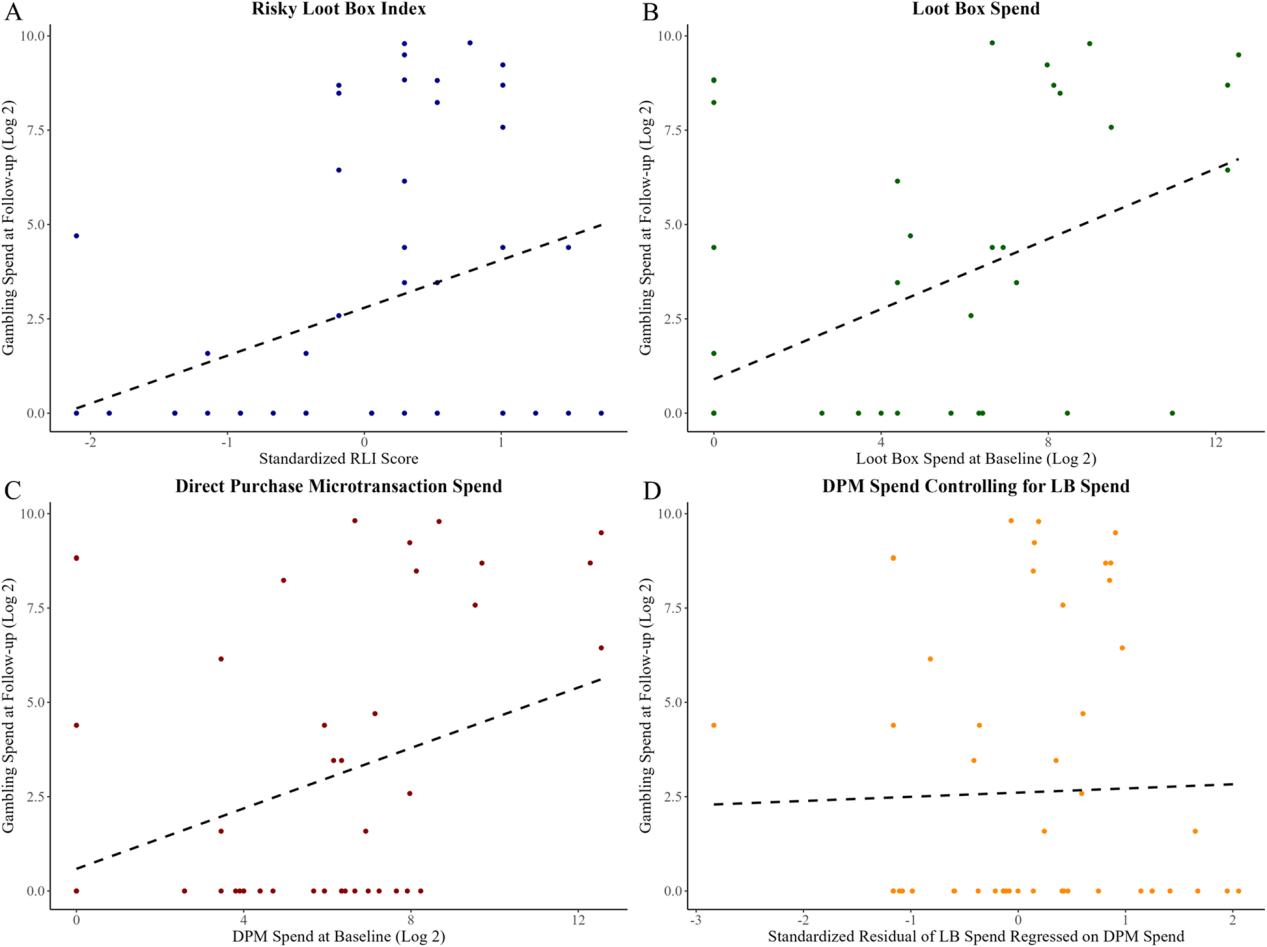
Linear regressions predicting gambling spend at follow-up. *Note*: Spending variables were log base 2 + 1 transformed to reduce positive skew. RLI scores were standardized. **A** shows the relationship between RLI scores and gambling spend at follow-up. **B** and **C** show the linear regression line for the effect of loot box and DPM spending on gambling spend at follow-up without controlling for either variable. **D** plots the standardized residual of loot box spending after regressing on DPM spending allowing for the visualization of the effect of DPMs when controlling for loot box spending

A similar hierarchical strategy was used for the linear models predicting 6-month gambling spend at follow-up. At step 1, when DPM spending was entered alone it significantly predicted the gambling spend at follow-up (B = 0.40, *p* = 0.005). However, when entered simultaneously with loot box spending at step 2, DPM spending did not significantly predict gambling spend (B = 0.04, *p* = 0.801). At step 2, baseline loot box spending significantly predicted gambling spending at follow-up, controlling for DPM spending (B = 0.44, *p* = 0.005). The RLI scores at baseline also significantly predicted gambling spend at follow-up (B = 1.27, *p* = 0.008). Table 4 summarizes the linear regression model output, and Fig. 1 displays the scatterplot and linear regression trends for each of the parameters in the linear regression models.

**Table 4:** Linear regressions predicting follow-up gambling spend (at T2) among baseline non-gambling participants

*12-Month Microtransaction Expenditure*						
**Variables – Step 1**	**B**	**98.75% CI**	**SE**	***β***	***t***	***p***-**value**
Constant	0.59	− 1.56, 2.73	0.83		0.708	.519
DPM spending	0.40	0.05, 0.75	0.14	0.38	2.93	.005
* R2/*Adj. *R2*	.142/.125					
* F*	8.59*					
**Variables—Step 2**	**B**	**98.75% CI**	**SE**	***β***	***t***	***p-*value**
Constant	0.77	− 1.25, 2.79	0.78		0.99	.328
DPM spending	0.04	− 0.42, 0.51	0.18	0.04	0.25	.801
Loot Box spending	0.44	0.04, 0.83	0.15	0.48	2.88	.005
* R2/*Adj. *R2*	.262/.233					
* F*	9.05*					
***Risky Loot Box Index***						
**Variables**	**B**	**98.75% CI**	**SE**	***β***	***t***	***p-*value**
Constant	2.79	1.57, 4.01	0.47		5.93	<.001
RLI score	1.27	0.40, 2.13	0.46	0.36	2.71	.008
* R2/*Adj. *R2*	.128/.111					
* F*	7.68*					

^*^Significance level of *p* ≤.0125 and 98.75% CI are required for the Bonferroni correction applied. Expense variables were log base 2 + 1 transformed to reduce positive skew. RLI scores were standardized

### Bayesian analysis

**Fig. 2:**
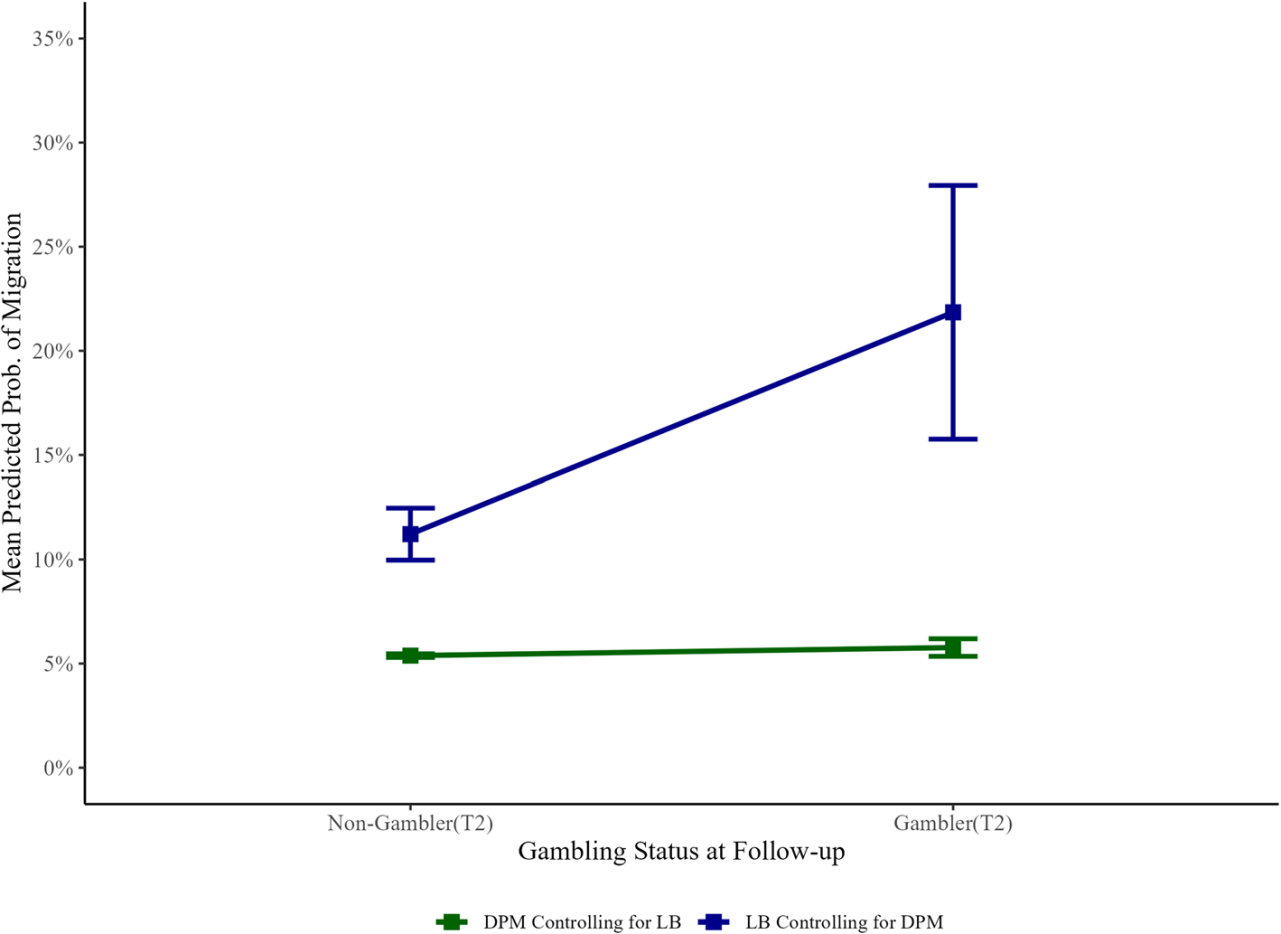
Mean predicted probability of migration from Bayesian logistic regression for DPM and loot box spending. *Note:* 98.75% CIs. Percentages display the mean predicted probability output from the Bayesian logistic regression of gambling migration based upon participants DPM spend while controlling for loot box expenditure, and participants loot box spend while controlling for DPM expenditure

A summary of model coefficients and Bayes factors are summarized in Table 5. In the logistic regression model predicting migration, the Bayes factors support the null hypothesis that baseline DPM spending is not associated with a greater likelihood of migration (OR = 1.02, BF_10_ = 0.72), and showed moderate support for the alternative hypothesis that baseline loot box spending is associated with greater likelihood of migration (OR = 1.27, BF_10_ = 7.46; see Fig. 2). For the RLI logistic model, the Bayes factors support the alternative hypothesis that baseline RLI scores are associated with increased risk of gambling migration at follow-up (OR = 1.70, BF_10_ = 3.19).

**Table 5:** Bayesian regressions predicting migration to gambling and gambling spend at follow up

Logistic Regression with baseline microtransaction spending as predictors						
**Variables**	**B**	**98.75% CI**	**SE**	**OR**	**98.75% CI OR**	**BF**_**10**_
Constant	− 3.71	− 5.13, − 2.36	0.56			
DPM spending	0.02	− 0.15, 0.20	0.07	1.02	0.86, 1.22	0.72
Loot Box spending	0.24	0.07, 0.40	0.07	1.27	1.07, 1.50	7.46
Logistic regression with baseline RLI scores as predictors						
**Variables**	**B**	**98.75% CI**	**SE**	**OR**	**98.75% CI OR**	**BF**_**10**_
Constant	− 2.06	− 2.50, − 1.63	0.17			
RLI	0.52	0.08, 0.97	0.18	1.70	1.09, 2.63	3.19
Linear regression with baseline microtransaction spending as predictors						
**Variables**	**B**	**98.75% CI**	**SE**			**BF**_**10**_
Constant	0.11	− 0.78, 0.99	0.35			
DPM spending	0.03	− 0.09, 0.15	0.05			1.54
Loot Box spending	0.22	0.10, 0.33	0.05			40.11
Linear regression with baseline RLI scores as predictors						
**Variables**	**B**	**98.75% CI**	**SE**			**BF**_**10**_
Constant	1.44	1.10,1.78	0.14			
RLI	0.56	0.21, 0.90	0.14			5.79

Model prior distributions were calculated based on data from Brooks & Clark [3]. Expense variables were log base 2 + 1 transformed to reduce positive skew. RLI scores were standardized

For the linear regressions on gambling spend, the Bayes factors indicated anecdotal evidence for the alternative hypothesis that baseline DPM spending is associated with increased follow-up gambling spending (B = 0.03, BF_10_ = 1.54), and strong evidence in favor of the alternative hypothesis that baseline loot box spending is associated with increased follow-up gambling spending (B = 0.22, BF_10_ = 40.11). The linear regression for the effect of RLI indicated moderate evidence in favor of the alternative hypothesis that baseline RLI would be associated with increased follow-up gambling spending. (B = 0.56, BF_10_ = 7.68).

## Discussion

This study aimed to establish further evidence of the prospective relationship between loot box use and conventional gambling behavior among young adults who play video games, by testing the replicability of results of Brooks & Clark [3] within a regional sample in Western Canada. In using online recruitment via regional Reddit groups, our sample size was smaller than intended and suffered further from substantial data loss (60%) in the cleaning stages. We adapted our analysis plan to the relatively small sample size by incorporating Bayesian tests to quantify strength of evidence for both the null and alternative hypotheses. Microtransaction spending and the Risky Loot Box Index (RLI) at baseline were tested as predictors of gambling initiation and gambling spend at follow-up. In the Bayesian analysis, loot box spending and RLI predicted gambling initiation in logistic regressions using informed priors from the Brooks & Clark data [3]. In the linear regressions, baseline loot box expenditure and RLI predicted gambling spend at follow-up in both the frequentist and Bayesian tests. Spending on DPMs did not predict gambling initiation in any set of models when controlling for loot box spending, underscoring the role of *randomized* rewards rather than spending per se in the associations with gambling behavior.

In our separation of gambling and non-gambling participants in the baseline survey, gambling participants showed higher levels of engagement with both randomized (i.e., loot boxes) and non-randomized (‘direct purchase’) microtransactions, and reported greater endorsement of buying and selling loot box items, replicating previous crowdsourced observations [3, 6], but in a local sample. It is notable that the current sample—recruited from Reddit communities and a local esports society—showed higher overall levels of microtransaction engagement, compared to the equivalent variables in Prolific samples. For example, the median loot box spend in the current study was $15.0 and $240.0 for the subset of non-gamblers and gamblers respectively, compared to $13.5 and $33.5 in Brooks & Clark [3]. This may indicate our Reddit sample was a more heavily involved group of gamers.

Microtransactions, including randomized in-game purchases, are now ubiquitous in video gaming [2, 10], and we observed that almost all our participants at the baseline assessment reported playing games that featured loot box or DPM mechanics, and that over two thirds of participants (gambling participants: 90.7%, non-gambling participants: 67.5%) had purchased loot boxes. The gambling participants at baseline spent 16 times more on loot boxes over the previous 12 months compared to non-gambling participants. These descriptive effects corroborate the wider literature around loot box engagement being more prominent among those who gamble [8, 12, 34].

The longitudinal analyses focused on the participants who reported no current gambling at the baseline survey. Within this group, our migration rate of 12.9% was close to the analogous value (11.3%) in Brooks & Clark [3], but with only 7 participants displaying migration in absolute terms, this compromised our statistical power in the two logistic regressions. As such, we failed to support the two hypotheses (H1 and H2) that baseline loot box purchasing and RLI score predict later initiation of gambling. Nevertheless, the frequentist linear regression analyses did support H3, that loot box spending significantly predicts later gambling spend, when controlling for DPM spending (Adjusted R2 = 0.233), and H4, that baseline RLI scores would predict later gambling spend (Adjusted R2 = 0.111).

Given the limited sample size of the current study, we conducted Bayesian equivalents of the preceding regression models with prior distribution for each parameter informed by the data from Brooks & Clark [3]. In addition to being able to quantify the degree of support for either the null or alternative hypothesis, Bayesian methods are also useful for refining predictions under a theory as evidence accumulates across studies of the same type, making it a particularly effective technique when comparing the results of a close replication to an original study [35, 36]. When the effects from Brooks & Clark [3] were used to inform the priors for the current analysis, we successfully replicated each of their effects. Specifically, per doubling of loot box expenditure at baseline, the odds of migrating to gambling status at follow-up was 27% (BF_10_ = 7.46), when controlling for DPM spend. Additionally, there was substantial evidence in favour of the hypothesis that baseline loot box spending predicts follow-up gambling spending in the analysis using informative priors (though with an attenuated effect size: B = 0.22, BF_10_ = 40.11), again controlling for the effect of DPM spending. For the Bayesian logistic RLI analysis, the odds of migration to conventional gambling increased by 70% per standard unit (BF_10_ = 2.95). Finally, the Bayesian linear regression firmly established the effect of RLI scores on follow-up gambling spend (B = 0.56, BF_10_ = 5.79). Altogether, the Bayesian regression analyses using informed priors provide further evidence that engagement with loot boxes increases the risk of conventional gambling at a 6-month follow-up [3, 17].

Some previous research indicates that spending on microtransactions in general (rather than loot boxes specifically), denotes the primary risk for increased financial harms from conventional gambling engagement [37, 38]. By using a two-step hierarchical regression strategy, we can distinguish the relative associations of spending on direct purchase forms versus randomized microtransactions (i.e., loot boxes). Specifically, the effect of DPM spending was not present in the model predicting follow-up gambling migration (OR = 1.02, BF10 = 0.72), and was negligible in the model predicting follow-up gambling spending (B = 0.03, BF10 = 1.54). This pattern of results supports the argument that the randomized nature of loot box mechanics is integral to the link between video game microtransaction spending and problem gambling severity [3, 39].

### Limitations

There are several limitations with the current study to consider. First, there is some nuance in operationalizing ‘current gambler’ status in our survey. By using the question “Do you currently gamble?”, a participant answering ‘no’ could still have earlier experiences with gambling. By defining group status with the alternative question “Have you ever gambled?” (see Table 1), participants who may have just gambled once in their lifetime would be classified as gamblers – which also reduces the sample size for the migration tests. Participants’ understanding of “currently” may also be subject to different interpretations, (e.g., past week, past month). Future studies could define a clearer timeframe for “current”, to improve precision. A related issue is that this item did not specify a focus on *monetary* gambling, meaning certain participants may have considered gambling for play money such as with valueless tokens or points when answering this question. There are notable differences in peoples experience and behaviour when gambling for real money compared to valueless play money (e.g., see Palmer et al., [40] for a scoping review on this subject), which may be relevant for understanding the link between conventional gambling and loot box use. For future studies, this ambiguity could be addressed by refining the item wording to focus on monetary gambling. A particular limitation with our dependent variables was that our longitudinal tests focus on gambling involvement but do not extend these effects to markers of problem gambling or gambling harm, which would likely require longer follow-up intervals. Additionally, we recognize that prospective designs allow one form of causal inference to be tested (i.e., temporal precedence or ‘Granger’ causality) and that other designs including experimental manipulations are needed to provide convergent evidence for causal pathways [39, 41].

A second set of limitations concerns our participant sampling approach. In sampling from the social media platform Reddit, there were consequences for data quality and a greater incidence of bots. We addressed this by adhering to strict data exclusion criteria, but this unfortunately led to a significant reduction in our sample size relative to Brooks & Clark 2023. Future researchers should take care to closely scrutinize the quality of data collected from social media platforms such as Reddit. It is also likely that recruiting from Reddit favored selection of a more engaged sample of gamers than might be seen on a crowdsourcing platform such as Prolific. This possibility is important in interpreting the generalizability of results; it is possible that the current effects are most likely in populations of higher intensity gamers. Future research in this area should aim to collect a more varied sample of gamers in terms of their gaming engagement to test this possibility. A further limitation of the current sample is the restriction to young adults. A primary concern for loot box related harms is the accessibility of these products to adolescents. We recruited young adults aged 19–25 given considerations around the legal gambling age, but future research could fruitfully conduct longitudinal studies in adolescents, and with longer follow-up windows. Most saliently, and as already discussed, our sample size was relatively small, due to our restrictive inclusion criteria being applied in a regional sample, but in combination with unexpectedly high data loss during cleaning to ensure data quality. We adapted our analysis plan to incorporate Bayesian statistics, and in our view, the evidential support for the migration effects in our Bayesian models highlights the value of this approach for other research testing replicability of effects on gambling and video gaming convergence.

## Conclusions

The chief aim of the current study was to determine whether the prospective effect of loot box engagement on later gambling initiation was reliable in a BC sample, to build knowledge at a provincial level. Acknowledging the mixed support in the frequentist analysis, the Bayesian analyses using informed priors provided a high level of support for the four hypotheses, including the specificity of these effects to randomized microtransactions over the non-randomized (DPM) format. Specifically, the observation that loot box spending predicted follow-up gambling over and above DPM spending in our multivariate hierarchical testing strategy emphasizes the role of ‘randomized’ rewards in the link between loot boxes and conventional gambling, as opposed to a mere willingness or capacity to spend as the underlying cause of this association. These findings highlight the public health relevance of video game loot boxes as an emerging gambling-like activity, and our cross-sectional and longitudinal observations indicate similar patterns in BC to other jurisdictions [3, 13, 17]. Recent prevalence data from BC indicate relatively high rates of gambling problems among adults who engage in online gambling (24%, compared to 9% among all those who gamble) [42]. In that survey, people at higher risk of problem gambling were also more likely to be male and younger, which are also known demographic predictors of engagement with gambling-like features of video games [43]. Further, a recent report on adolescent health in BC observed that 20% of the 11–16 year old sample had purchased a loot box in the past 12-months [44]. Given these regional considerations, healthcare providers should be attentive to the risks associated with gambling-like activities in video games. We recommend greater screening for financial harm among patients seeking treatment for excessive video gaming, and the incorporation of psychological elements in the treatment of gambling disorder into corresponding therapies for loot box mechanics in video games.

## Data Availability

Data used in the reported analyses along with the analysis code for the Bayesian regressions is available at the following link: https://osf.io/47yub/?view_only=ee47c9171ba84fdebd63f38ce8e70375.

## Abbreviations

DPM: Direct purchase microtransaction
RLI: Risky loot box index

## Footnotes

1 See Availability of Data and Materials for informed prior analysis script.
